# High-mechanical-frequency characteristics of optomechanical crystal cavity with coupling waveguide

**DOI:** 10.1038/srep34160

**Published:** 2016-09-30

**Authors:** Zhilei Huang, Kaiyu Cui, Guoren Bai, Xue Feng, Fang Liu, Wei Zhang, Yidong Huang

**Affiliations:** 1 Department of Electronic Engineering, Tsinghua National Laboratory for Information Science and Technology, Tsinghua University, Beijing, 100084, China

## Abstract

Optomechanical crystals have attracted great attention recently for their ability to realize strong photon-phonon interaction in cavity optomechanical systems. By far, the operation of cavity optomechanical systems with high mechanical frequency has to employ tapered fibres or one-sided waveguides with circulators to couple the light into and out of the cavities, which hinders their on-chip applications. Here, we demonstrate larger-centre-hole nanobeam structures with on-chip transmission-coupling waveguide. The measured mechanical frequency is up to 4.47 GHz, with a high mechanical *Q*-factor of 1.4 × 10^3^ in the ambient environment. The corresponding optomechanical coupling rate is calculated and measured to be 836 kHz and 1.2 MHz, respectively, while the effective mass is estimated to be 136 fg. With the transmission waveguide coupled structure and a small footprint of 3.4 μm^2^, this simple cavity can be directly used as functional components or integrated with other on-chip devices in future practical applications.

Optomechanical crystals (OMCs)^1^, also known as phoxonic crystals^2^, are artificial materials that simultaneously control photon and phonon by periodic patterning of nanostructures. They have attracted great attention recently for their ability to realize cavity optomechanical systems, which are platforms to study or realize strong photon-phonon interaction^3^.

Increasing the mechanical frequency of OMCs is crucial in both fundamental science study and practical applications. For instance, the high mechanical frequency phonon is more resistant to the environmental disturbance and benefits the experiments in quantum regime^4^. Besides, side-band resolved condition is easier to be achieved with high mechanical frequency^5^, which is related to many kinds of experiments, such as cooling^6^, optomechanically induced transparency^7^, and coherent wavelength conversion^8^. High mechanical frequency also extends the corresponding microwave frequency range^9^ and benefits the performances of many functional optomechanical devices, such as modulators^10^, transducers^11^, and sensors^12^.

As phonon manipulation is realized by OMCs with sub-micro patterns, short acoustic wavelength, corresponding to the high mechanical frequency, can be achieved in OMCs. For one-dimensional structure, a mechanical frequency of 5.9 GHz has been predicted in heterostructure^3^ and 4.2 GHz has also been demonstrated by a corrugated structure with the complete bandgap^13^. Recently, a mechanical frequency tunable structure has been proposed with the ability of achieving a phononic mode of 10.2 GHz^14^.

On the other hand, how to couple light into the OMC cavities is another important issue. Vertically coupled tapered fibres are mainly used for this purpose^1^,^13^,^15^. And the technique of employing one-sided waveguides with circulators has also been demonstrated^16^. Even though these coupling methods are available for preliminary measurements, they are hardly compatible with integrated on-chip functional devices, such as the integrated quantum processing^17^, the bio-sensing^18^, or the optical nonreciprocity^19^. Coupling light into OMC cavities with separate waveguides has been demonstrated recently^20^,^21^,^22^. Although this method enables the on-chip integration of OMC cavities, the corresponding optical transmission spectra are dip-type, i.e., at the optical resonant wavelength, where light is strongly phase modulated by the mechanical motion^23^, the optical transmission power is lowest. So the coupling scheme suffers a high transmission loss. Additionally, since both the OMC cavities and the coupling waveguides are fragile suspended structures, the light coupling between them is unstable.

Here, we experimentally demonstrate the structure proposed in our previous work^24^, so called larger-centre-hole OMC cavities, with integrated transmission-coupling waveguides based on silicon-on-insulator (SOI) chip around telecom wavelength. High mechanical frequencies spanning from 4.4 to 5.7 GHz with different geometric parameters of the structures is demonstrated. Moreover, the mechanical *Q*-factor measured in the ambient environment is as high as 1.4 × 10^3^, and the cavity is ultra-compact with the length of only 6.1 μm, corresponding to the footprint less than 3.4 μm^2^. The optical transmission spectrum of the structure is peak-type, i.e., the structure possesses the highest optical transmission power at the resonant wavelength. And due to the nanobeam cavity intrinsically integrated with the transmission-coupling waveguide, the optical coupling is stable and unaffected by the displacement of the waveguide. The compact cavities with light coupled by integrated transmission waveguides can be directly used as functional components or integrated with other on-chip devices in future practical applications.

## Results

### Theoretical analysis

**Figure 1:**
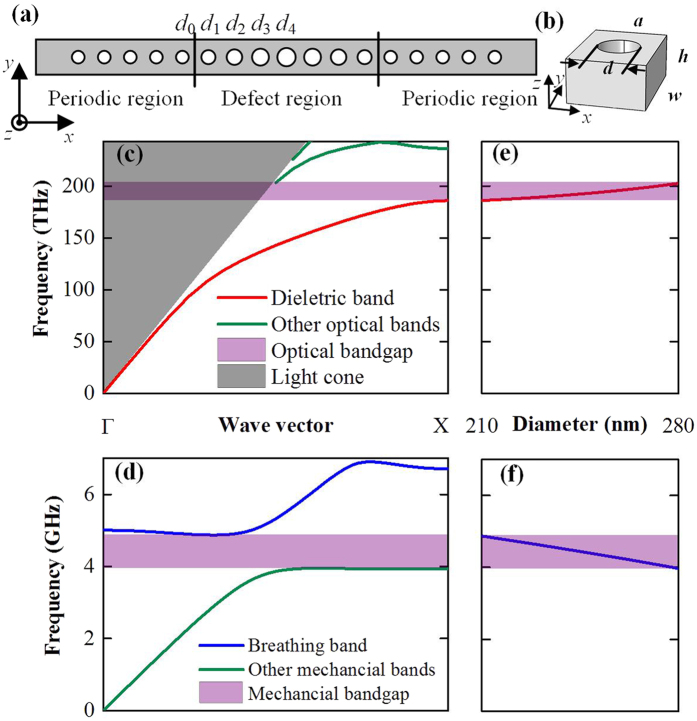
(**a**) The plan-view schematic of the larger-centre-hole OMC nanobeam cavities. (**b**) The unit cell constituting the structure. (**c**,**d**) The TE optical bands and *y*-, *z*-symmetric mechanical bands of the periodic unit cell of the example structure. The red line represents the dielectric band for optical mode and the blue line represents the breathing band for the mechanical mode. The purple regions represent the optical bandgap and mechanical bandgap, respectively. The grey region in (**c**) represents the light cone. (**e**,**f**) The frequency variation of the X point of the dielectric band and the lowest point of the breathing band as the diameter of air hole extends from 210 to 280 nm.

The plan-view schematic of the larger-centre-hole nanobeam optomechanical cavity is shown in Fig. 1(a). The unit cell constituting the structure is given in Fig. 1(b), which is a silicon block with an air hole. The geometry of the unit cell can be characterized by the height (*h*), the pitch (*a*), the width (*w*), and the diameter of the air hole (*d*_0_). The periodic regions at both sides of the cavity are constituted by the identical unit cell set, while the defect region is constituted by seven symmetric distributed unit cells. The height of the structure is 220 nm for a standard SOI chip. Since the diameter of the holes in the defect region varies linearly from side to centre, the geometry of the whole structure is determined by pitch (*a*), width (*w*), the diameter of the air hole (*d*_0_), and the extended ratio (*ER*), which is the ratio between the diameter of the central hole and that of the periodic hole, i.e. *d*_1_/*d*_0_ = 0.25(*ER* − 1) + 1, *d*_2_/*d*_0_ = 0.5(*ER* − 1) + 1, *d*_3_/*d*_0_ = 0.75(*ER* − 1) + 1, *d*_4_/*d*_0_ = *ER*.

The mechanical frequency increases with the reduction of acoustic wavelength, which can be achieved by reducing the size of the solid object. However, an object with too small size is hard to support optical mode. Thus it is hard to improve the mechanical frequency of an optomechanical system while ensuring optical mode’s existing. To analyse how the proposed structures simultaneously form both optical and mechanical cavities, we calculate the optical and mechanical bands, here the unit cell with (*a*, *w*, *d*_0_) = (365, 556, 210) nm is selected for analysis. Figure 1(c) shows the optical bands of TE symmetric mode of the periodic structure formed by this unit cell. The first band indicated with the red line is the dielectric band as the optical field concentrates in silicon rather than air for these modes. It can be seen that, for this specific unit cell, it possesses optical bandgap between 186 to 204 THz. Figure 1(d) shows the mechanical bands of the *y*-, *z*-symmetric mode of the periodic structure formed by this unit cell with a bandgap between 3.95 to 4.87 GHz. The modes of the second band indicated by the blue line are “breathing modes”, named by its mode profile^1^. Figure 1(e,f) show frequency variations at the X point of the dielectric band and the lowest point of the mechanical band as the diameter of the unit cell extends from 210 to 280 nm. During this extension, both the optical dielectric mode and the mechanical breathing mode move into the bandgap formed by the origin unit cell. Thus, acting as mirrors, the periodic regions simultaneously confine the optical and the mechanical modes in the defect region. As shown in Fig. 1(e,f), the optical and the mechanical modes can simultaneously exist in the bandgap with a large diameter range (from 210 to 280 nm), optomechanical cavity can be formed with *ER* = *d*_4_/*d*_0_ from 1 to 1.33 for this case. Therefore, the optomechanical cavity can be formed with *ER* in a large range.

**Figure 2:**
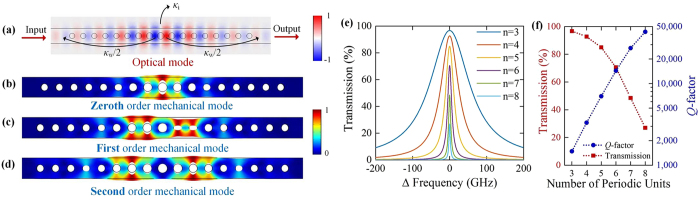
(**a**) The electric *y* component of the optical mode of the example structure with loss mechanism illustrated. (**b**–**d**) The displacement of zeroth, first and second order mechanical mode. The mechanical frequencies of them are 4.40, 4.56 and 4.75 GHz, respectively. The zeroth and second order modes are *x*-symmetric, while the first order mode is *x*-asymmetric. (**e**) The calculated optical transmission spectra with different number of periodic units. (**f**) The calculated optical transmission efficiency and the optical *Q*-factor with varying the number of periodic units.

In order to analyse the optical and mechanical mode of the entire structure, we take *ER* = 1.253 for simulation, which is the value of our fabricated cavity. Figure 2(a,b–d) show the optical mode and the mechanical modes. For the mechanical modes, the frequencies are 4.40 GHz, 4.56 GHz, and 4.75 GHz, accordingly. Here, the first order mechanical mode is *x*-asymmetric as shown in Fig. 2(c), which means that the mechanical variations of the two sides affect the optical mode equally but reversely. Thus, the first mode will not couple with the optical mode^24^, and we just consider the zeroth and second order mechanical modes.

As both the optical and the mechanical modes are concentrated in silicon material within the small defect region, the interaction strength between the optical and the mechanical modes can be enhanced in the proposed structure. Based on our studies, a high optomechanical coupling rate can be obtained in this structure^24^. Specifically, the optomechanical coupling rate ( *g*/2π) between the optical and zeroth order mechanical modes is 836 kHz. Since the centre of the mechanical mode is occupied with larger holes, the volume of the vibrating part is quite small. Here, the effective mass is used to quantitatively describe the localization of the mechanical mode. For the zeroth mode, the effective mass is as small as 136 fg. Such a small effective mass could benefit the precision for transducing^11^ and sensing^12^,^25^.

Since the transmitted light carries both the optical and mechanical information, extracting the transmitted light efficiently is crucial for a practical integrated functional component. As illustrated in Fig. 2(a), part of the light in the cavity leaks through the periodic structure to the coupling waveguide and the other part of the light radiates into the surrounding environment due to the phase mismatch introduced by the defect region^26^. The energy loss caused by the two mechanisms respectively is referred as waveguide coupling loss and intrinsic loss and the decay rate of them are denoted as *κ*_w_ and *κ*_i_. As derived by Joannopoulos, *et al*.^27^ the optical transmission at the resonant frequency (*T*) can be expressed as


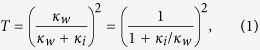


which is related to the ratio between *κ*_i_ and *κ*_w_. We calculate the transmission spectra of the cavities with different number of periodic units, which is shown in Fig. 2(e), and summarize the transmission efficiency and the optical *Q*-factor in Fig. 2(f). It should be noted that the intrinsic optical quality is determined by the defect region, which can be analysed by the calculation of intrinsic *Q*-factor using Finite Difference Time Domain (FDTD) method. To further improve the intrinsic *Q*-factor, the defect region can be carefully designed to further suppress the optical radiation loss. For this purpose, more tapered holes in the defect region could be added and optimized by deterministic method^28^ or parameters swapping method^29^. Then, with a well-designed defect region, the waveguide coupling can be reduced and fixed by the simulation of the entire structure. Here, the number of holes in the periodic structure is chosen to balance the performance of the light transmission and the total optical *Q*-factor. As shown in Fig. 2(f), with the increment of the number of periodic units, the transmission efficiency decreases, while the optical *Q*-factor increases. This tendency agrees well with Eq. (1). To balance the transmission efficiency and the *Q*-factor, we choose five unit cells in the periodic region. At this point, a high transmission efficiency over 85% can be achieved with a *Q*-factor of 7.0 × 10^3^. In summary, such lager-centre-hole structures can simultaneously confine the optical mode around telecom wavelength and the mechanical mode around 5 GHz with a strong interaction between them. The effective mass is predicted as small as 136 fg. Moreover, the high transmission efficiency indicates that this simple lager-centre-hole cavity can easily couple with the integrated transmission waveguides.

### Measurement results

**Figure 3:**
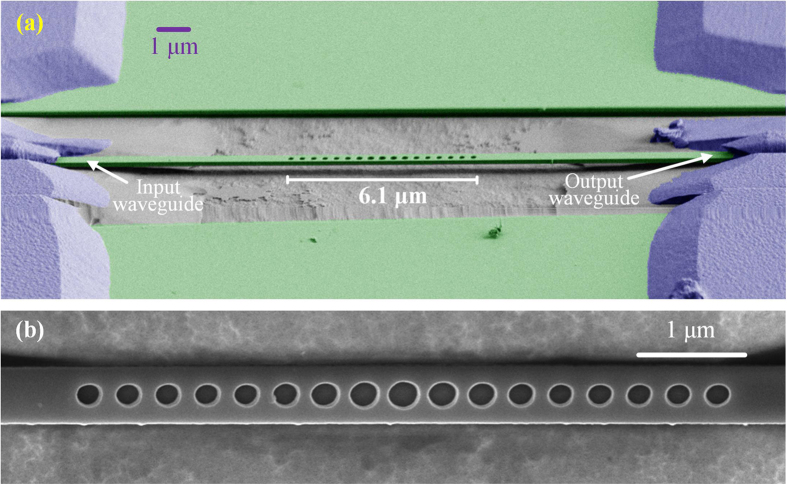
(**a**) The coloured SEM image of the oblique view of the fabricated structure. The device layer of silicon, including the cavity and the waveguide, is coloured with green and the deposited silica is coloured with blue. (**b**) The SEM image of the top view of central cavity.

Figure 3(a) shows the oblique view of the coloured scanning electron microscope (SEM) image of the fabricated structure and Fig. 3(b) shows the top view SEM image of the central cavity. The device layer of silicon, including the cavity and the waveguide, is coloured with green and the deposited silica is coloured with blue, while the colour of the other parts, including the buried oxide layer of silica and the substrate silicon, remains grey. The geometric parameters of our fabricated structure are (*a*, *w*, *d*_0_, *ER*) = (365 nm, 556 nm, 211 nm, 1.253), as that discussed in the theoretical analysis. The length of the cavity is only 6.1 μm. It should be noted that the input and output coupling waveguides are buried under silica and can be directly connected to other on-chip components. As the image of the nanobeam obviously separates from the “shadow” of the cantilever on the substrate, this picture shows the suspended structure clearly.

**Figure 4:**
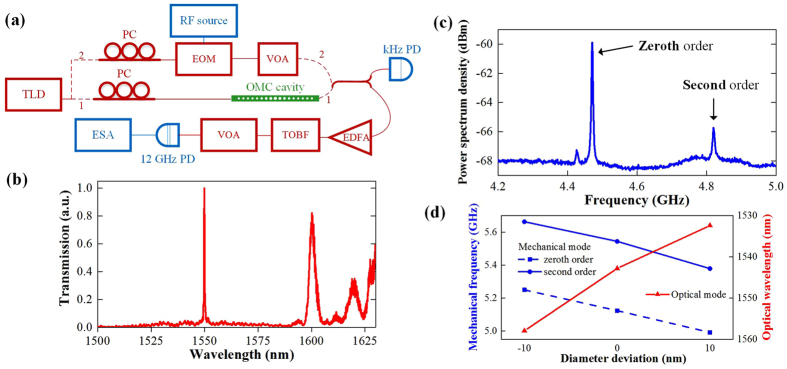
(**a**) The measurement setup. TLD, tunable laser diode; PC, polarization controller; PD, photodetector; EDFA, erbium-doped fibre amplifier; TOBF, tunable optical bandpass filter; VOA, viable optical attenuator; ESA, electrical spectrum analyser. (**b**,**c**) The normalized optical transmission spectrum and the power spectrum density of the first structure. (**d**) Wavelength and mechanical frequency variation as the diameter of the air holes of the cavity structure changes. The structures parameters are (*a*, *w*, *d*, *ER*) = (372 nm, 495 nm, 184 nm, 1.365). Around this cavity parameter, cavities with all air holes extending or shrink 10 nm in diameter are also fabricated.

Based on the fabricated structure, we measure the optical transmission spectrum and the mechanical power spectrum density. Figure 4(a) shows the schematic of the measurement system in the ambient environment. In experiments, the system is firstly connected as route 1 to measure the optical and the mechanical properties of the OMC cavity. The light from the tunable laser diode (TLD) is first adjusted to TE polarization for efficient coupling. Then, the TE polarization light is coupled into and out of the transmission waveguides by end-fire coupling method with tapered and lensed fibres. A 50/50 fibre coupler splits the output light into two ports. One port is connected to a kHz photodetector (PD), and the optical transmission spectrum can be acquired from this PD by sweeping the wavelength of the TLD whose wavelength is set near the resonant wavelength of the cavity to measure the mechanical properties. The other port is connected to an erbium-doped fibre amplifier (EDFA), filtered by a tunable optical bandpass filter (TOBF) to reduce the amplified spontaneous emission noise, and adjusted by a viable optical attenuator (VOA) before received by a PD with a bandwidth of 12 GHz. The output electric signal from PD is measured by an electrical spectrum analyser (ESA). Since the optical resonant frequency shifts with the cavity vibrating, the optical transmission power of the chip is modulated by the mechanical motion. Thus, the mechanical information can be obtained by measuring the power spectral density of the electrical output of the PD with the electrical spectrum analyser.

The measured normalized optical transmission spectrum is shown in Fig. 4(b). This spectrum indicates an optical mode at 1550 nm with a *Q*-factor of 6.0 × 10^3^. Based on the measurement, we can get the difference of the *Q*-factor between the measured result (6.0 × 10^3^) and the simulated prediction (7.0 × 10^3^), which is mainly caused by the scattering loss due to the fabrication imperfection. Thus, the scattering loss can be reduced, which is about one-sixth of the radiation loss. By setting the wavelength of the TLD near the resonate frequency, the power spectral density measured by the electrical spectrum analyser is shown in Fig. 4(b). The two peaks at 4.47 and 4.82 GHz indicate the zeroth and second order mechanical mode, respectively, which agrees well with the simulated frequency of 4.40 and 4.75 GHz. The Lorentzian fitting of the peak near 4.47 GHz indicates a mechanical *Q*-factor of 1.4 × 10^3^ in the ambient environment (room temperature and atmospheric pressure). It should be noted that, though the mechanical mode is confined by only five periodic unit cells with phononic bandgap for *y*-, *z*-symmetry, the mechanical *Q*-factor of the cavity is comparable with the nanobeam structure with complete phononic bandgap^13^, where the mechanical *Q*-factor is ~2.0 × 10^3^ in the ambient environment.

The optomechanical coupling rates (*g*/2π) between the optical mode and the two mechanical modes are calculated to be 836 kHz and 396 kHz, respectively, which affects the intensity of the power spectrum density. Assuming the *Q*-factors of the two mechanical modes are roughly equal, the intensity of the power spectrum density is proportional to *g*^2^/*n*_c_^4^. Here, *n*_c_ is the phonon number of the mode, which can be calculated by Bose-Einstein distribution, i.e. 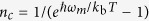. Thus, by calculating the two expressions mentioned above, the intensity ratio of these two modes should be 4.8, which agrees well with the experimental measured value of 4.7 as shown in Fig. 4(c).

To quantitatively measure the optomechanical coupling rate of the zeroth order mechanical mode, we connect the optical system as route 2 as shown in Fig. 4(a) to calibrate the strength of mechanical signal generated by the OMC cavity. The principle of the calibration technique employed here is similar to that used by Gavartin, *et al*.^30^ The calibration signal we used is intensity modulated since the high-speed PD detects the optical intensity signal. The VOA in route 2 is used to ensure the output optical power stays the same after switching the optical route. By comparing the electrical power detected by the ESA, the optomechanical coupling rate can be estimated as (Supplementary Section S1)


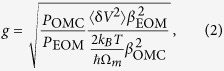


where *P*_OMC_ and *P*_EOM_ are the power of the high frequency signal generated by the OMC cavity and the calibration electro-optic modulator (EOM), respectively, <δ*V*^2^> is the square of the effective value of the driving signal voltage of the EOM, *β*_OMC_ and *β*_EOM_ are the optical intensity modulation factor of the OMC cavity and the EOM, respectively. Here, the modulation factor of the EOM is the ratio of the light intensity modulated by the EOM with unit driving voltage. Similarly, the modulation factor of the OMC cavity is the ratio of the light intensity modulated by the OMC cavity with unit frequency shift. *T* is the temperature of the measurement environment, Ω_m_ is the angular frequency of the mechanical mode, and *k*_B_ and 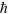 are the Boltzmann and Planck constant, respectively. Based on Eq. (2), the optomechanical coupling rate between the optical mode and the zeroth order mechanical mode is estimated to be 1.2 MHz (Supplementary Section S2). The discrepancy between the measured and simulated result is similar with that of other OMC nanobeam cavities based on silicon^31^. Both the uncertainties of the silicon photo-elastic coefficients in simulation^31^ and the imprecision in measurement may contribute to the discrepancy.

To verify the proposed structure can maintain an optomechanical cavity with quite structure deviations, cavities with different geometry parameters are fabricated and measured. Figure 4(d) plots the variation of the resonant optical wavelength and the mechanical frequency as the diameters of the air holes change. Here, we fabricate structures with (*a*, *w*, *d*_0_, *ER*) = (372 nm, 495 nm, 184 nm, 1.365). This structure supports optical mode at 1543 nm and higher frequency mechanical modes at 5.12 GHz and 5.54 GHz. Around this cavity parameter, cavities with all air holes extending or shrinking 10 nm in diameter are also fabricated. Though these cavities are fabricated with larger roughness, which deteriorates the optical *Q*-factor and mechanical signal, the optical and mechanical mode can still be detected using the system in Fig. 4(a) in the ambient environment. As analysed in Fig. 1(e,f), extension of the air holes results in decreasing of the mechanical frequency and increasing of the optical frequency (corresponding to decreasing of wavelength), which agrees with the measurement results shown in Fig. 4(d) and verifies the structure can simultaneously support optical and mechanical mode with a large range of geometry parameters deviations.

## Discussion

In conclusion, we have demonstrated a transmission waveguide coupled OMC with the optical mode at telecom wavelength and a high mechanical frequency of 4.4 GHz. The mechanical *Q*-factor measured in the ambient environment is 1.4 × 10^3^, which is comparable with the nanobeam structure with complete phononic bandgap. The corresponding optomechanical coupling rate for the zeroth order mode is calculated and measured to be 836 kHz and 1.2 MHz, respectively. And the effective mass of the fabricated structure is calculated to be 136 fg. With a quite deviation of the structure geometry, the proposed structures are robust and still maintain optomechanical cavities with the mechanical frequencies spanning from 5.0 to 5.7 GHz. Moreover, this cavity is ultra-compact, and the length and the footprint are only 6.1 μm and 3.4 μm^2^, respectively. Therefore, with the transmission waveguide coupling structure, the proposed simple cavities can be directly used as functional components or integrated with other on-chip devices in future practical applications.

## Method

### Simulation

The optical transmission spectrum is calculated by Finite Difference Time Domain (FDTD) method. The photonic band structures are calculated by plane wave expansion (PWE) method. The phononic bands and the optical and mechanical modes, are achieved by finite element method (FEM). For calculating the optomechanical coupling rate, the optical and mechanical modes are simultaneously solved in the same mesh system, so that the set of the domain and the surface is consistent for the calculation of photoelastic effect and moving boundary effect^31^.

### Fabrication

The structures are fabricated as follows. The cavity and waveguide patterns are first defined by electron beam lithography (EBL) and transferred to the device layer of SOI chips by inductively coupled plasma (ICP) etching. Then 600-nm-thick silica is deposited on the top by using plasma enhanced chemical vapour deposition (PECVD) and the chips are back-grinded to 100-μm-thick for future cleavage. After that, 10-μm-width strip windows above the cavities are formed by aligned ultraviolet (UV) lithography. Then the buried and deposited oxide layers beneath and above the cavities are removed by wet etching with buffered hydrofluoric acid (BHF) to form the suspended structures. Finally, the chips are cleaved and prepared for testing.

## Additional Information

**How to cite this article**: Huang, Z. *et al*. High-mechanical-frequency characteristics of optomechanical crystal cavity with coupling waveguide. *Sci. Rep.* **6**, 34160; doi: 10.1038/srep34160 (2016).

## Supplementary Material

Supplementary Information
