# Supplementary material for: High-mechanical-frequency characteristics of optomechanical crystal cavity with coupling waveguide
Source: Sci Rep. 2016 Sep 30;6:34160. doi: 10.1038/srep34160 (PMC5043188; doi:10.1038/srep34160)
Supplement: Supplementary Information [file srep34160-s1.pdf]

Supplementary Information

High-mechanical-frequency characteristics of optomechanical crystal cavity with coupling waveguide

Zhilei Huang, Kaiyu Cui*, Guoren Bai, Xue Feng, Fang Liu, Wei Zhang, and Yidong Huang

Department of Electronic Engineering, Tsinghua National Laboratory for Information Science and Technology, Tsinghua University, Beijing, 100084, China

*kaiyucui@tsinghua.edu.cn

S1. Expression for of optomechanical coupling rate measurement

The optomechanical coupling rate for an optomechanical system is defined as

$$g = \frac{\partial \omega_{\mathrm{o}}}{\partial x} x_{\mathrm{zpf}}, \tag{S1}$$

where ω_{o} is the optical angular frequency of the cavity, x is the displacement of the mechanical resonator and x_{zpf}, which equals to $\sqrt{\hbar / 2m_{\mathrm{eff}}\Omega_{\mathrm{m}}}$, is the displacement of zero-point fluctuation of the mechanical resonator. Consequently, the intensity of mechanical vibration caused optical frequency shift is related with g. Quantitatively, the square of the optical resonant frequency fluctuation can be expressed as[1]

$$\left\langle \delta\omega_{\mathrm{o}}^{2} \right\rangle = 2n_{\mathrm{c}} g^{2}, \tag{S2}$$

where n_{c} is the phonon occupation number of the interested mechanical mode. In this work, the mechanical motion is mainly excited by thermal environment, thus

$$n_{\mathrm{c}} = \frac{k_B T}{\hbar \Omega_m}, \tag{S3}$$

where T is the temperature of the measurement environment, Ω_{m} is the angular frequency of the mechanical mode and k_{B} and $\hbar$ are the Boltzmann and Planck constant, respectively.

As illustrated in Fig. S1, the optical frequency shift causes the transmission power vibration. So the optical

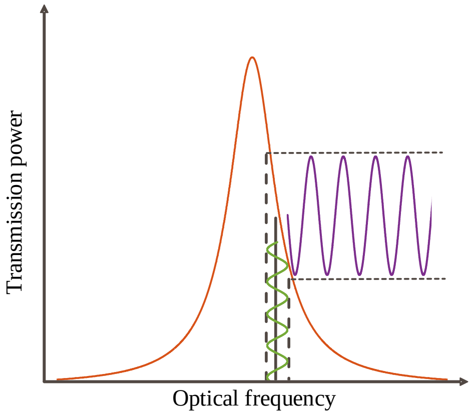

Figure S1. Illustration of the relation between the optical frequency shift and the transmission power vibration. The red line is the optical transmission curve of the cavity. As the input laser frequency set near the resonant frequency (the solid black line), the vibration of the optical frequency (illustrated as green oscillatory line) causes the vibration of the optical transmission (illustrated as purple oscillatory line). The amplitude of the of optical transmission vibration is determined by the amplitude of the frequency shift as well as the slope of the transmission curve at the setting frequency.

resonant frequency fluctuation can be determined by measure the vibration of optical transmission power. We define β_{OMC} as the optical intensity modulation factor of the optomechanical crystal (OMC) cavity, i.e., $\delta I / I = \beta_{\mathrm{OMC}}\, \delta\omega$, where I is the optical intensity of the transmitted light. This leads to the relation between the optical intensity fluctuation and the optical resonant frequency fluctuation as

$$\frac{\left\langle \delta I_{\mathrm{OMC}}^2 \right\rangle}{I^2} = \beta_{\mathrm{OMC}}^2 \left\langle \delta\omega_{\mathrm{o}}^2 \right\rangle. \tag{S4}$$

As shown in the section S2, $<\delta I^2>$ is calibrated by an electro-optic intensity modulator (EOM). We define β_{EOM} as the optical intensity modulation factor of the EOM, i.e., $\delta I / I = \beta_{\mathrm{OMC}}\, \delta V$, where δV is the voltage of the electric signal applied on the EOM. So the optical intensity fluctuation caused by the EOM is

$$\frac{\left\langle \delta I_{\mathrm{EOM}}^2 \right\rangle}{I^2} = \beta_{\mathrm{EOM}}^2 \left\langle \delta V^2 \right\rangle. \tag{S5}$$

The power of the signal around the interested frequency detected by the ESA is proportional to the square of the optical intensity fluctuation if the average optical intensity is fixed, i.e.

$$\frac{P_{\mathrm{OMC}}}{P_{\mathrm{EOM}}} = \frac{\left\langle \delta I_{\mathrm{OMC}}^2 \right\rangle}{\left\langle \delta I_{\mathrm{EOM}}^2 \right\rangle}. \tag{S6}$$

By applying Eq. (S2-S5) to Eq. (S6), the optomechanical coupling rate can be expressed as

$$g = \sqrt{\frac{P_{\text{OMC}} \left\langle \delta V^2 \right\rangle \beta_{\text{EOM}}^2}{P_{\text{EOM}} \dfrac{2k_B T}{\hbar \Omega_m} \beta_{\text{OMC}}^2}}. \tag{S7}$$

S2. Optomechanical coupling rate measurement

The experiments for measuring the optomechanical coupling rate was conducted 1 year later than that only measuring the optical and mechanical frequency and *Q*-factor. Thus, the performances of the fabricated structure deteriorate, especially the mechanical *Q*-factor. Consequentially, only the zeroth order mode can be observed. However, as the optomechanical coupling rate is depended on the profiles of optical and mechanical modes, it should vary little, like the optical and mechanical frequency.

Figure S2(a) shows the output of ESA while light passing through the OMC cavity and the EOM, respectively. The RF source generating a 4.40 GHz signal with root-mean-square (RMS) voltage of 5 mV. The resolution bandwidth (RBW) of the ESA is set to be 300 kHz during the measurement. This RBW is much smaller than the mechanical spectrum width of the OMC cavity but much larger than that of signal generated by the RF source. As the output of the swept-tuned type ESA is the power filtered by a Gaussian bandpass filter, the RF power of the EOM signal detected by the ESA is the peak value (i.e. P_{EOM} = -51.93 dBm) while that of the OMC cavity signal needs integration. Figure S2(b) shows the measurement data of ESA for the OMC cavity in linear coordinate as well as the Lorentzian fitting curve. Consequently, the RF power detected by the ESA for the OMC cavity can be expressed as

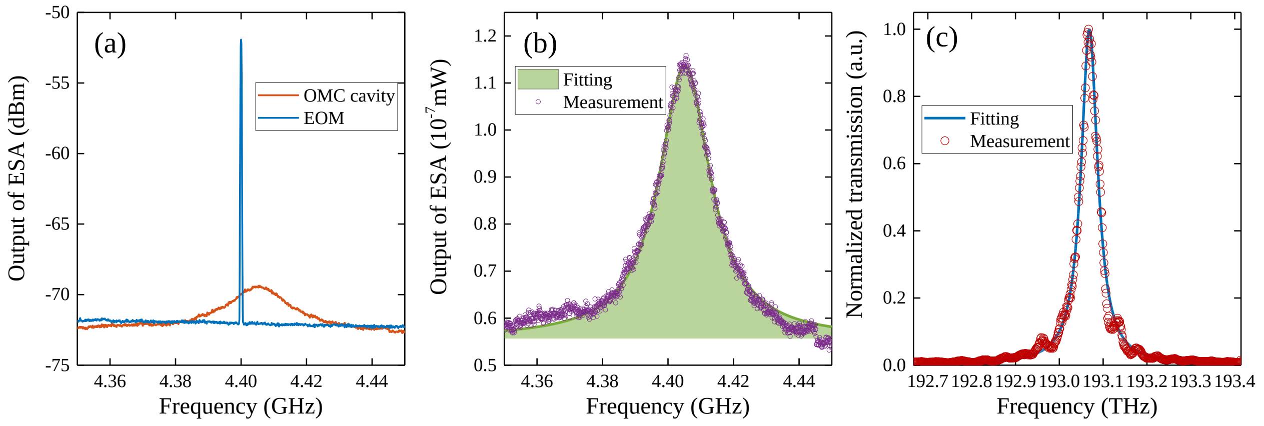

Figure S2. (a) The output of the ESA when the OMC cavity or the EOM is connected in the system. (b) The measurement and fitting data of the output of ESA when the OMC cavity is connected in the system. (c) The measured and fitted data of the normalized optical transmission spectrum of the OMC cavity.

$$P_{\mathrm{OMC}} = \frac{Area}{1.064RBW}, \tag{S8}$$

where *Area* is the area between the Lorentzian fitting curve and the background noise, i.e. the green area shown in Fig S2(b), and the coefficient of 1.064 is originated from the ratio between the area and the width of the Gaussian curve. For this measurement, P_{OMC} = -52.20 dBm is obtained.

To determine the β_{OMC}, we analyse the optical transmission spectrum of the cavity. In frequency domain, the optical transmission spectrum is a Lorentzian curve, i.e.

$$T(\omega) = T_0 \frac{\kappa^2}{4(\omega - \omega_{\mathrm{o}})^2 + \kappa^2}, \tag{S9}$$

where T_0 is the maximum transmission and κ is the total decay rate of the cavity, which equals to the full width at half maximum (FWHM). The β_{OMC} is maximized to be $2/\kappa$, $44/2\pi$ THz^{-1} for this OMC cavity, when frequency detuning $|\omega\text{-}\omega_{\mathrm{o}}|$ equals to $\kappa/2$. During the measurement of mechanical spectrum of the OMC cavity, we set the wavelength of the laser at the blue detuning point. The β_{EOM} of the EOM we used at 4.4 GHz is about 0.58 V^{-1}.

Based on the conditions provided above, the optomechanical coupling rate ($g/2\pi$) is estimated to be 1.2 MHz.

Reference

1. Gorodetsky, M. L., Schliesser, A., Anetsberger, G., Deleglise, S. & Kippenberg, T. J. Determination of the vacuum optomechanical coupling rate using frequency noise calibration. *Opt. Express* **18,** 23236–46 (2010).
